# Supplementary material for: Drosophila as a Model of Unconventional Translation in Spinocerebellar Ataxia Type 3
Source: Cells. 2022 Apr 4;11(7):1223. doi: 10.3390/cells11071223 (PMC8997593; doi:10.3390/cells11071223)
Supplement: Supplementary file 1 [file cells-11-01223-s001.zip › cells-1657254-supplementary.pdf]

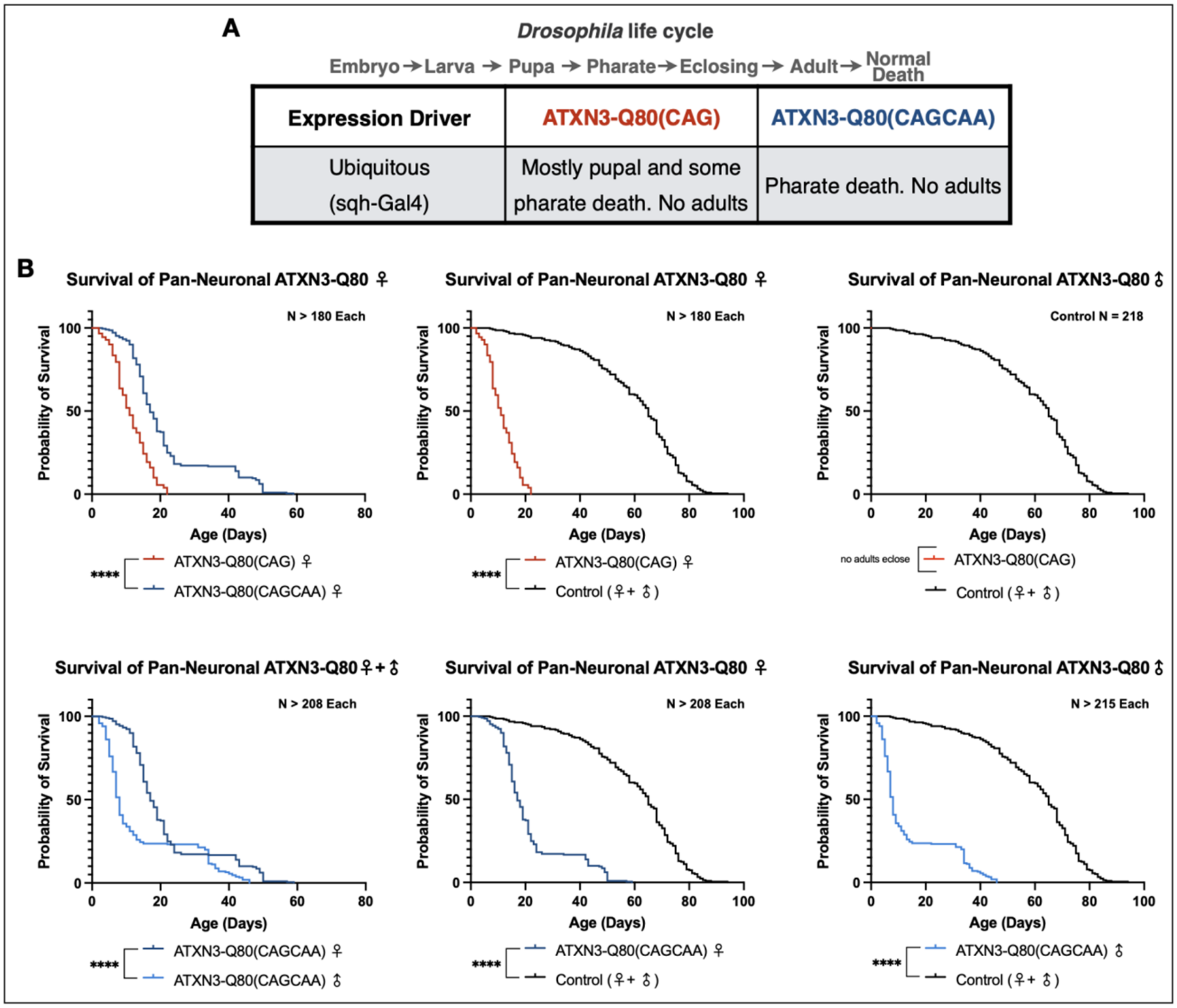

Figure S1. ***Ubiquitous expression longevity data and individual comparisons from survival analysis in figure 2***

(A) Ubiquitous expression of ATXN3-Q80(CAG) and ATXN3-Q80(CAGCAA) individually was developmentally lethal in flies. Observations of developmental deaths in both lines based on the fly life cycle (shown at top of figure) showed ATXN3-Q80(CAG) flies dying primarily at earlier developmental stages than flies ubiquitously expressing ATXN3Q80(CAGCAA). These observations were confirmed with survival analysis in tissue-specific expression models shown in figure 2. (B) Individual comparisons of all pan-neuronal expressing fly lines shown in figure 2E. "*": p<0.0332; "**": p<0.0021; "***": p<0.0002; "****": p<0.0001. p-values from log-rank test. No adults eclosed in the glial expression analysis in figure 2F, thus no individual comparisons were made.

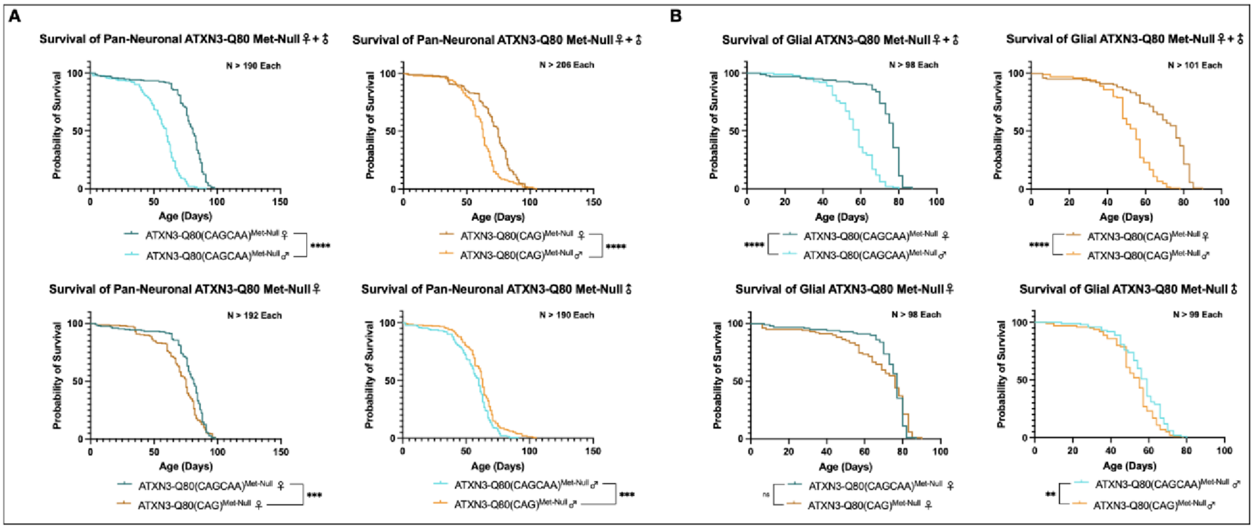

Figure S2. ***Individual comparisons from survival analysis in figure 6***

(A) Individual comparisons of all pan-neuronal expressing fly lines shown in figure 6A. "*": p<0.0332; "**": p<0.0021; "***": p<0.0002; "****": p<0.0001. p-values from log-rank test. (B) Individual comparisons of all glial expressing fly lines shown in figure 6C. "ns": non-statistically significant; "*": p<0.0332; "**": p<0.0021; "***": p<0.0002; "****": p<0.0001. pvalues from log-rank test.

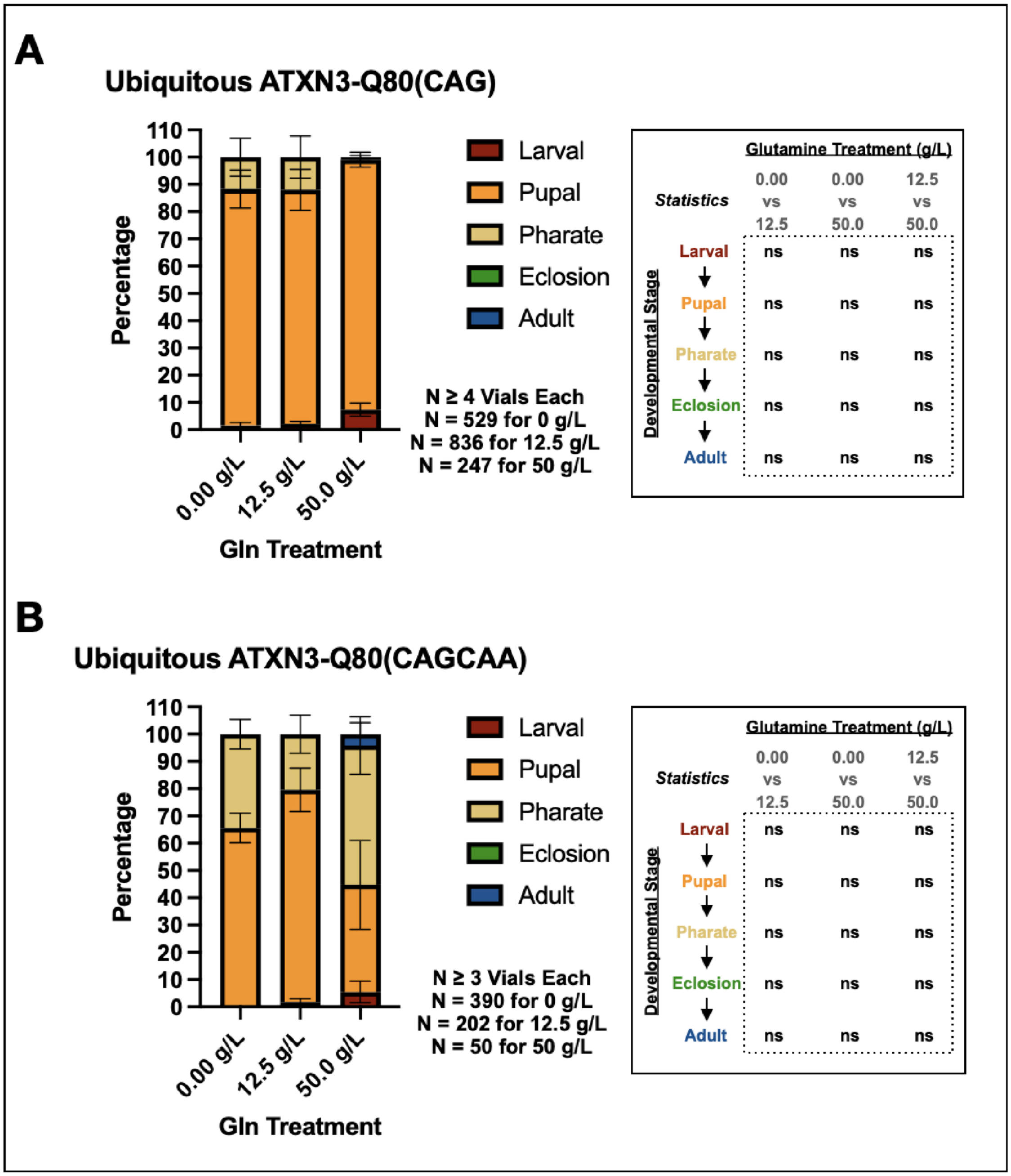

Figure S3. ***Developmental deaths of ATXN3-Q80-expressing flies with glutamine supplementation***

(A) Developmental death tracking based on the percentage of fly deaths at each developmental stage in flies ubiquitously expressing ATXN3-Q80(CAG) in food with 0.00, 12.5, or 50.0 g/L glutamine supplementation. Driver was sqh-Gal4. Each liter of food made accounted for 100 fly vials, with each vial then receiving an additional 0.000, 0.125, or 0.500 g/L of glutamine respectively. Means of percentages at each stage +/- SEM. "ns": non-statistically significant. p-values of all comparisons shown to the right of graph from RM two-way ANOVA with Geisser-Greenhouse correction and Tukey's multiple comparison test. n ≥ 3 vials each. (B) Developmental death tracking based on percentage of fly deaths at each developmental stage in flies ubiquitously expressing ATXN3-Q80(CAGCAA) in food with 0.00, 12.5, or 50.0 g/L glutamine supplementation in food. Means of percentages at each stage +/- SEM. "ns": nonstatistically significant. p-values of all comparisons shown to the right of graph and from RM two-way ANOVA with Geisser-Greenhouse correction and Tukey's multiple comparison test. n ≥ 3 vials each.

Figure S4: Nucleotide sequences for Panel 5A

Met-Null CAGCAA Sequence

GAATTCATTTAGGAATAGTAGGAGTCCATCTTCCACGAGAA
ACAAGAAGGCTCACTTTGTGCTCAACATTGCCTGAATAACT
TATTGCAAGGAGAATATTTTAGCCCTGTGGAATTATCCTCAA
TTGCACATCAGCTGGATGAGGAGGAGAGGTAGAGATAGGC
AGAAGGAGGAGTTACTAGTGAAGATTATCGCACGTTTTTAC
AGCAGCCTTCTGGAAATTAGGATGACAGTGGTTTTTTCTCT
ATTCAGGTTATAAGCAATGCCTTGAAAGTTTGGGGTTTAGAA
CTAATCCTGTTCAACAGTCCAGAGTATCAGAGGCTCAGGAT
CGATCCTATAAATGAAAGATCATTTATATGCAATTATAAGGAA
CACTGGTTTACAGTTAGAAAATTAGGAAAACAGTGGTTTAA
CTTGAATTCTCTCTTGACGGGTCCAGAATTAATATCAGATAC
ATATCTTGCACTTTTCTTGGCTCAATTACAACAGGAAGGTTA
TTCTATATTTGTCGTTAAGGGTGATCTGCCAGATTGCGAAGC
TGACCAACTCCTGCAGTAGATTAGGGTCCAACAGTAGCAT
CGACCAAAACTTATTGGAGAAGAATTAGCACAACTAAAAGA
GCAAAGAGTCCATAAAACAGACCTGGAACGAGTGTTAGAA
GCAAATGATGGCTCAGGATAGTTAGACGAAGATGAGGAGG
ATTTGCAGAGGGCTCTGGCACTAAGTCGCCAAGAAATTGA
CTAGGAAGATGAGGAAGCAGATCTCCGCAGGGCTATTCAG
CTAAGTTAGCAAGGTAGTTCCAGAAACATATCTCAAGATTAG
ACACAGACATCAGGTACAAATCTTACTTCAGAAGAGCTTCG
GAAGAGACGAGAAGCCTACTTTGAAAAACAGCAACAGAAA
CAACAGCAACAGCAACAGCAACAGCAACAGCAACAGCAA
CAGCAACAGCAACAGCAACAGCAACAGCAACAGCAACAG
CAACAGCAACAGCAACAGCAACAGCAACAGCAACAGCAA
CAGCAACAGCAACAGCAACAGCAACAGCAACAGCAACAG
CAACAGCAACAGCAACAGCAACAGCAACAGCAACAGCAA
CAGCAACAGCAACAGCAACAGCAACAGCAACAGCAACAA
CAGCAAGGGGACCTATCAGGACAGAGTTCACATCCATGCG
AAAGGCCAGCCACCAGTTCAGGAGCACTTGGGAGCGATC
TCGGCGATGCTTAGAGCGAAGAAGACTAGCTTCAGGCAG
CTGTCACCTAGTCTTTGGAAACTGTCAGAAACGATTTGAAA
ACAGAAGGAAAAAAATACCCATACGATGTTCCAGATTACGC
TTAATAAAATGGAACAAAAACTTATTTCTGAAGAAGATCTGT
GGTAAGCCTATCCCTAACCCTCTCCTCGGTCTCGATTCTAC
GTAATAATCTAGA

Met-Null CAGCAG Sequence

GAATTCATTTAGGAATAGTAGGAGTCCATCTTCCACGAGAA
ACAAGAAGGCTCACTTTGTGCTCAACATTGCCTGAATAACT
TATTGCAAGGAGAATATTTTAGCCCTGTGGAATTATCCTCAA
TTGCACATCAGCTGGATGAGGAGGAGAGGTAGAGATAGGC
AGAAGGAGGAGTTACTAGTGAAGATTATCGCACGTTTTTAC
AGCAGCCTTCTGGAAATTAGGATGACAGTGGTTTTTTCTCT
ATTCAGGTTATAAGCAATGCCTTGAAAGTTTGGGGTTTAGAA
CTAATCCTGTTCAACAGTCCAGAGTATCAGAGGCTCAGGAT
CGATCCTATAAATGAAAGATCATTTATATGCAATTATAAGGAA
CACTGGTTTACAGTTAGAAAATTAGGAAAACAGTGGTTTAA
CTTGAATTCTCTCTTGACGGGTCCAGAATTAATATCAGATAC
ATATCTTGCACTTTTCTTGGCTCAATTACAACAGGAAGGTTA
TTCTATATTTGTCGTTAAGGGTGATCTGCCAGATTGCGAAGC
TGACCAACTCCTGCAGTAGATTAGGGTCCAACAGTAGCAT
CGACCAAAACTTATTGGAGAAGAATTAGCACAACTAAAAGA
GCAAAGAGTCCATAAAACAGACCTGGAACGAGTGTTAGAA
GCAAATGATGGCTCAGGATAGTTAGACGAAGATGAGGAGG
ATTTGCAGAGGGCTCTGGCACTAAGTCGCCAAGAAATTGA
CTAGGAAGATGAGGAAGCAGATCTCCGCAGGGCTATTCAG
CTAAGTTAGCAAGGTAGTTCCAGAAACATATCTCAAGATTAG
ACACAGACATCAGGTACAAATCTTACTTCAGAAGAGCTTCG
GAAGAGACGAGAAGCCTACTTTGAAAAACAGCAGCAGAAA
CAGCAGCAGCAGCAGCAGCAGCAGCAGCAGCAGCAGCA
GCAGCAGCAGCAGCAGCAGCAGCAGCAGCAGCAGCAGC
AGCAGCAGCAGCAGCAGCAGCAGCAGCAGCAGCAGCAG
CAGCAGCAGCAGCAGCAGCAGCAGCAGCAGCAGCAGCA
GCAGCAGCAGCAGCAGCAGCAGCAGCAGCAGCAGCAGC
AGCAGCAGCAGCAGCAGCAGCAGCAGCAGCAGCAGCAG
CAGCAGCAGCAGGGGGACCTATCAGGACAGAGTTCACAT
CCATGCGAAAGGCCAGCCACCAGTTCAGGAGCACTTGGG
AGCGATCTCGGCGATGCTTAGAGCGAAGAAGACTAGCTTC
AGGCAGCTGTCACCTAGTCTTTGGAAACTGTCAGAAACGA
TTTGAAAACAGAAGGAAAAAAATACCCATACGATGTTCCAG
ATTACGCTTAATAAAATGGAACAAAAACTTATTTCTGAAGAA
GATCTGTGGTAAGCCTATCCCTAACCCTCTCCTCGGTCTC
GATTCTACGTAATAATCTAGA
